# Abnormal Electrophysiological Motor Responses in Huntington’s Disease: Evidence of Premanifest Compensation

**DOI:** 10.1371/journal.pone.0138563

**Published:** 2015-09-25

**Authors:** Lauren M. Turner, Rodney J. Croft, Andrew Churchyard, Jeffrey C. L. Looi, Deborah Apthorp, Nellie Georgiou-Karistianis

**Affiliations:** 1 Research School of Psychology, College of Medicine, Biology, & Environment, Australian National University, Canberra, Australia; 2 School of Psychology & Illawarra Health & Medical Research Institute, University of Wollongong, Wollongong, Australia; 3 School of Psychological Sciences, Faculty of Medicine, Nursing and Health Sciences, Monash University, Victoria, Australia; 4 Calvary Health Care Bethlehem Hospital, Caulfield, Victoria, Australia; 5 Research Centre for the Neurosciences of Ageing, Academic Unit of Psychiatry and Addiction Medicine, Australian National University Medical School, Canberra Hospital, Canberra, Australia; 6 Melbourne Neuropsychiatry Centre, Department of Psychiatry, Faculty of Medicine, Dentistry and Health Sciences, University of Melbourne, Melbourne, Australia; University of Pennsylvania Perelman School of Medicine, UNITED STATES

## Abstract

**Background:**

Huntington's disease (HD) causes progressive motor dysfunction through characteristic atrophy. Changes to neural structure begin in premanifest stages yet individuals are able to maintain a high degree of function, suggesting involvement of supportive processing during motor performance. Electroencephalography (EEG) enables the investigation of subtle impairments at the neuronal level, and possible compensatory strategies, by examining differential activation patterns. We aimed to use EEG to investigate neural motor processing (via the Readiness Potential; RP), premotor processing and sensorimotor integration (Contingent Negative Variation; CNV) during simple motor performance in HD.

**Methods:**

We assessed neural activity associated with motor preparation and processing in 20 premanifest (pre-HD), 14 symptomatic HD (symp-HD), and 17 healthy controls. Participants performed sequential tapping within two experimental paradigms (simple tapping; Go/No-Go). RP and CNV potentials were calculated separately for each group.

**Results:**

Motor components and behavioural measures did not distinguish pre-HD from controls. Compared to controls and pre-HD, symp-HD demonstrated significantly reduced relative amplitude and latency of the RP, whereas controls and pre-HD did not differ. However, early CNV was found to significantly differ between control and pre-HD groups, due to enhanced early CNV in pre-HD.

**Conclusions:**

For the first time, we provide evidence of atypical activation during preparatory processing in pre-HD. The increased activation during this early stage of the disease may reflect ancillary processing in the form of recruitment of additional neural resources for adequate motor preparation, despite atrophic disruption to structure and circuitry. We propose an early adaptive compensation mechanism in pre-HD during motor preparation.

## Introduction

Huntington’s disease (HD), a monogenetic, neurodegenerative disease, causes progressive motor, cognitive, and behavioural impairment [1]. Symptom onset occurs in the 4^th^ decade of life; however, there is robust evidence of striatal and cortical atrophy up to 20 years prior to diagnosis [2–8]. Despite early neuropathological changes, individuals retain normal functions. Subtle cognitive and motor changes (e.g., tapping precision) have been shown to commence up to a decade prior to onset but progress at a slower rate than atrophy [8–9]. Specifically, observable motor impairment has been highlighted during more complex tasks,[10] which may implicate recruitment of ancillary regions to offset dysfunction in motor control. There is a growing interest in understanding the underlying brain mechanisms that enable individuals to function normally in the context of early degenerative processes. Structural degradation may be mitigated by neural compensation, which could support optimal performance during early stages of HD [3–5, 11–13]. Electroencephalography (EEG) provides a means of investigating functional impairment at the neuronal (ensemble) level, and offers an important opportunity to further investigate the mechanisms driving compensation [14]. In this context, EEG measures may also offer a new avenue for functional biomarker discovery where postsynaptic neural activity could be evaluated during early drug development. In this study, we focus on the temporal properties of neural ensemble activity characterising various elements of motor control.

Neuropathological changes, such as striatal volume loss, may have minimal functional consequences due to efferent and reciprocal connections between brain regions [15]. That is, networks requiring coordination from multiple groups of neurons may maintain function despite localised damage. Previous functional magnetic resonance imaging (fMRI) studies have identified premanifest changes to neural networks, including reduced functional connectivity in cortico-striatal networks implicated in motor control [5, 16–19]. Recently, decreased synchrony of sensorimotor networks was found to correlate with motor imprecision during speeded self-tapping, and to precede cognitive impairment in dorsal networks [16]. This suggests that an impairment of motor circuitry may represent an early signature of disease progression. Cortical shifts, such as concurrent brain activation, may enable premanifest individuals to compensate effectively for early deficits in motor control, while still cognitively intact [11].

Investigations of functional connectivity in motor control support recruitment of ancillary circuity to supplement motor performance. For example, in symptomatic individuals (symp-HD), reduced primary motor activation has been accompanied by parietal overactivation [20–22] and in presymptomatic individuals (pre-HD) by excessive thalamo-cortical activation [23]. In particular, Klöppel and colleagues [13] identified increased compensation in pre-HD individuals, involving flexible recruitment of premotor and parietal areas dependent on the pace and complexity of motor sequences. In studies with pre-HD, no between-group differences have been identified in task performance, further suggesting the motor system is adept at re-organising itself according to task demands [13].

Elicited from EEG, movement-related potentials (MRPs) provide a measure of cortical activity associated with movement preparation and execution [24]. The Readiness Potential (RP) represents movement preparation and is decreased in amplitude in movement disorders such as Parkinson’s disease [24–25]. Reduced RP components have also been previously reported in symp-HD, compared with controls [12, 26–27]; however, in pre-HD no RP differences have been detected despite increased inhibition in the hemisphere ipsilateral to dominant hand which may reflect compensatory mediation by GABAergic transmission [11–12]. In premotor activation, the Contingent Negative Variation (CNV) has also been found to be significantly reduced in symp-HD [28]; to date no CNV studies have been conducted in pre-HD.

In this investigation, we aimed to evaluate neural motor mechanisms in both pre-HD and symp-HD, compared with healthy controls, using electrophysiological measures (i.e., RP and CNV). In doing so, we aimed to evaluate the neural compensatory hypothesis first proposed by Beste and colleagues [12] in relation to ancillary motor control during a simple finger tapping task.

## Materials and Methods

### Participants

Forty-four participants were recruited for this study, consisting of 14 HD individuals (11 males; symp-HD), 20 prodromal individuals (12 males; pre-HD), and 17 healthy controls (9 males), age-matched to the pre-HD group. The EEG recording session entailed two tasks and participants with response amplitudes greater than -25 μV or excessive muscular artifacts were excluded. In Task 1 (cued and self-paced tapping), 7 participants were excluded, 3 pre-HD (3 males), and 4 symp-HD (3 males); in Task 2 (sensorimotor integration), 3 participants were excluded including 1 pre-HD (1 male), and 2 symp-HD (1 male). Participant demographics and clinical data for the overall sample are provided in Table 1. Pre-HD and symp-HD participants underwent genetic testing to confirm gene status and estimate CAG repeat length. Both HD groups of were assessed by a neurologist (A.C.) and underwent the Unified Huntington’s Disease Rating Scale (UHDRS)[29] as a measure of motor severity. HD individuals with an UHDRS TMS (Total Motor Score) of < 5 were included in the pre-HD group and those with scores of > 5 in the symp-HD group (consistent with TRACK-HD)[6]. Formal ethics approval was granted by Monash University, with written informed consent was obtained from all participants. All clinical investigation was conducted according to the principles expressed in the Declaration of Helsinki.

**Table 1:** Demographic data for participants included in analyses of RP and CNV task. Standard deviations (SD) provided in parentheses.

	Controls(n = 17)	Pre-HD(n = 20)	Symp-HD(n = 14)
Gender (M:F)	9:8	12:8	11:3
Age	41.00 (11.32)	41.11 (10.88)	58.64 (10.31)^a^ ** ^b^ **
Education (yrs)	12.59 (2.10)	12.36 (2.31)	11.14 (2.71)
CAG repeat length	-	41.53 (2.50)	42.21 (1.97)
CAG-index	-	238.08 (101.88)	346.08 (68.26)^b^ **
Probability of Diagnosis in 5 years	-	0.17 (0.20)	-
Illness Duration	-	-	4.07 (3.83)
DBS	-	239.39 (100.66)	387.82 (111.54)
UHDRS	-	0.63 (1.16)	22.29 (10.62)
IQ estimate	104.88 (27.76)	112.92 (6.03)	109.70 (7.03)
BDI-II	2.12 (3.02)	7.16 (9.98)	8.14 (8.38)
Trails B	61.30 (16.33)	67.73 (21.60)	166.79 (77.63)^a^ ** ^b^ **
Speeded Tapping	186.25 (55.32)	218.61 (25.32)	362.45 (114.51)^a^ ** ^b^ **
HVLT Total Recall	28.24 (3.87)	25.16 (4.89)	15.86 (7.01)^a^ ** ^b^ **
HVLT Delayed Recall	10.06 (1.89)	8.89 (2.13)	5.50 (3.23)^a^ ** ^b^ **
HVLT % Retention	88.46 (15.37)	88.78 (10.80)	78.17 (33.03)
HVLT Recognition Discrimination Index	11.53 (1.46)	9.89 (1.41)^a^ *	7.93 (2.43)^a^ ** ^b^ *

CAG, cytosine-adenine-guanine; CAG-index, [CAG_n_(# of CAG repeats)– 35.5]multiplied by age; probability of onset in 5-years; DBS, Disease Burden Score (CAG-35.5)*age; UHDRS, motor subscale score, Unified Huntington’s Disease Rating Scale (pre-HD, UHDRS <5; symp-HD, UHDRS ≥5); IQ (NART: National Adult Reading Test 2^nd^ Edition); BDI-II, Beck Depression Inventory—second edition; Trails B, Trail Making Test Two-Target-Switch; HVLT, Hopkins Verbal Learning Test. Independent samples t-test for differences between groups;

* *p* < .05,

** *p* < .01;

^a^ significantly different from controls,

^b^ significantly different from pre-HD.

Neurocognitive testing included a speeded tapping task to assess motor performance [30], Trails B to assess executive functioning [31], and the Hopkins Verbal Learning Task (HVLT) to assess memory functioning [32]. Depressive symptoms were assessed using the Beck Depression Inventory-II (BDI-II)[33].

### Procedures

#### EEG tasks

As part of a larger study, participants completed a series of electrophysiological tasks. In order of presentation, these consisted of an EOG calibration task [34], a cued and self-paced tapping task (Task 1), sensorimotor integration task (Go/No-Go paradigm; Task 2), working memory rehearsal, auditory and cognitive processing (oddball paradigm), face emotion processing, sustained attention task, with practice trials immediately prior to each task. Only the tapping and sensorimotor integration tasks are reported here.

#### Cued and Self-Paced Tapping (Task 1)

Participants performed a sequential tapping task on a response pad (Version 4.0; Compumedics, Neuroscan, TX, USA) using their right index finger. They were required to alternate between two buttons (left and right), at a rate of one every four seconds. The task comprised two Conditions (cued and self-paced). In the cued Condition, accompanying visual cues were provided by a square appearing on either the left or right of the screen. The cues were presented for 1000 ms, once every four seconds, for a period of 24 s. Participants were instructed to respond on the same side as the indicated cue as quickly as possible. In the self-paced Condition, participants continued to press alternating buttons at the same rate in the absence of cues. The two Conditions alternated for a total of 8 minutes, resulting in a total of 60-cued and 60 self-paced button presses in the task.

#### Sensorimotor Integration (Task 2)

Participants were presented with a 500 ms blue light flash (S1), followed 2.5 s later (fixed interval) by an ‘X’ or ‘Y’ visual cue (S2). S2-No-Go (Y) cues remained on screen for 1.9 s; S2-Go (X) cues remained on screen until a response. The ‘X’ stimulus was designated as a Go stimulus, requiring a motor response; the ‘Y’ stimulus was designated as a No-Go stimulus, indicating response should be withheld. Presentation of ‘X’ and ‘Y’ cues was varied randomly between the left and right sides of the screen, with location (of the ‘X’) indicating a corresponding button press of the ipsilateral key as quickly as possible using the right index finger. A total of 90 trials were performed, with the No-Go stimulus occurring in 20% of trials. The inter-trial intervals were randomly varied between 2500 and 4000 ms.

#### Electrophysiological Recording

Presentation of stimuli and recording of behavioural responses were controlled by Stim2 (Version 4.0; Compumedics, Neuroscan, TX, USA). EEG data were recorded and processed using Scan 4.1 (Compumedics, Neuroscan, TX) software. A 40-channel Lycra EEG cap with embedded tin surface electrodes was used, following the international 10/20 system. The EEG was referenced to a point midway between Cz and Pz, with a ground electrode located midway between Fz and FPz. Impedances were below 10 kΩ for all electrodes at the start of the recording. Eye movements (EOG) were measured for subsequent EOG correction, with electrodes placed above and below the left eye, and on the outer canthus of each eye. EEG and EOG signals were amplified using a NuAmps 40-channel DC amplifier (Compumedics, Neuroscan, TX) with a digital bandpass filter at 0.15–100 Hz, and sampled at 1000 Hz.

### Data Analysis

#### Electrophysiological Data. Readiness Potential (RP)—Cued and Self-Paced Tapping (Task 1)

Offline RP data were EOG-corrected [34], and bandpass filtered using a 20 Hz (48 dB roll-off) to 100 Hz (12 dB roll-off) zero phase shift filter. The epoch segment comprised the period -2998 ms before to 1000 ms following movement execution (button press), with subsequent baseline correction (relative to -2500 to -2000 ms), artifact rejection procedures (±150μV; excluding EOG channels), and further removal of visually determined contaminated trials. Epochs were then averaged separately for each electrode site and Condition (i.e., cued and self-paced). As RP amplitude was maximal at Cz, analysis was restricted to this site. Participants with less than 15 epochs for a Condition were excluded from further analysis. The relative amplitude and latency of the RP respectively was computed using peak-to-peak values comprised of the difference between the negative peak occurring at -50 to 100 ms, and the positive peak 100 to 300 ms post response. Owing to inter-individual variability in activation, peak-to-peak values were used to provide a more accurate reflection of elevation from baseline during the RP than peak amplitudes, which may be affected by a range of factors including motivation during the task.

#### Contingent Negative Variation—Sensorimotor Integration (Task 2)

CNV data were EOG-corrected [34], and bandpass filtered using a 0.03 to 35 Hz (24 dB roll-off) zero phase shift filter consistent with previous literature.[28] Data were epoched to the period of -3500 to 1000 ms relative to S2 (Go-No-Go) cue presentation and baseline corrected (relative to -3500 to -3000 ms). Artifact rejection procedures were automated (±150μV; excluding EOG channels), with additional rejection of contaminated trials based on visual inspection. Epochs were averaged separately for each electrode site; both Go and No-Go trials were included in the grand average. CNV Amplitudes were measured as maximum amplitudes obtained in the periods 550–750 ms following the presentation of S1 (early CNV; eCNV), and in the 200 ms prior to the onset of S2 respectively (late CNV; lCNV)[28]. Peak-to-peak values were used to better account for inter-individual variation and degree of activation across the entire CNV profile (early and late peaks), rather than amplitude values. Values were calculated using the difference between the maximum amplitude obtained during the eCNV, and that of the lCNV.

#### Statistical Analysis

For each task separately, each behavioural measure (reaction time, inter-tap intervals, inter-tap interval variability), RP (amplitude, latency) and CNV component (early, late, difference) was examined separately (dependent variable) using a mixed design ANCOVA, with group (symp-HD; pre-HD; controls) the between-subjects factor and age a covariate. For Task 1, RP analyses contained a within-subjects factor Condition (cued; self-paced). For Task 2, early and late CNV values were initially taken at Cz.Subsequent exploratory analysis of sites Fz and Pz was based on prior studies [24], and was included to investigate possible abnormal activation in these sites [13, 20–23]. Bonferroni adjustments were used to control for multiple comparisons in all post-hoc testing. Pearson partial correlations (controlling for age) were used to examine the relationship between electrophysiological variables and clinical measures of disease progression for pre-HD and symp-HD groups.

## Results

### Behavioural

Behavioural results for Task 1 and Task 2 are presented in Tables 2 and 3 respectively. For Task 1 (cued and self-paced tapping), there was a significant main effect of Group for tapping variability (F (2, 40) = 6.691, *p* = .003), with symp-HD more variable than control (*p* = .003) and pre-HD (*p* = .008) groups. For Task 2 (sensorimotor integration) there was a significant main effect of Group for reaction time (*F* = 11.360, *p* < .001), with symp-HD (1306.66 ± 324) slower than controls (750.97 ± 203, *p* < .001) and pre-HD (779.11 ± 243.62, *p* < .001) groups. There was no difference in Go/No-Go errors between groups, with all groups responding accurately to each condition.

**Table 2:** Electrophysiological and behavioural results for participants in the Tapping Task 1 (cued and self-paced Conditions). Amplitude and latency by at Cz, averaged across groups. Standard deviations (SD) provided in parentheses.

	Controls(n = 17)	Pre-HD(n = 17)	Symp-HD(n = 10)
**Behavioural (Tapping)**			
***Time between taps***			
Cued	4.47 (.035)	4.42 (.058)	4.53 (.080)
Self-paced	4.12 (.118)	4.08 (.147)	4.17 (.359)
***Variability***			
Cued	.20 (.015)	.28 (.036)	.47 (.091)^a^ ** ^b^ *
Self-paced	.67 (.108)	.69 (.125)	1.45 (.249)^a^ ** ^b^ **
**Electrophysiological (Amplitude/Latency)**			
***Cued***			
Peak Amplitude	-6.128 (6.57)	-5.255 (4.73)	-2.866 (7.96)
Relative Amplitude	-9.118 (4.22)	-7.483 (3.53)	-4.016 (1.91) ^a^ ** ^b^ **
Relative Latency	-164.23 (70.51)	-193.41 (59.24)	-190.80 (81.37)
***Self-paced***			
Peak Amplitude	-8.615 (5.47)	-7.132 (5.94)	-5.638 (8.74)
Relative Amplitude	-10.28 (4.95)	-7.883 (2.81)	-4.961 (2.70) ^a^ ** ^b^ **
Relative Latency	-158.11 (52.19)	-174.94 (55.48)	-226.40 (63.43)

Relative amplitude and latency are computed based on the difference in peak values between the negative peak occurring -50 to 100ms prior to the motor response, and the positive peak 100 to 300 ms post response. Peak amplitude represents the negative peak occurring around time of response (-150 to 200 ms). Independent samples t-tests used for differences between groups,

* *p* < .05,

** *p* < .01.

^a^ significantly different from controls,

^b^ significantly different from pre-HD.

**Table 3:** Electrophysiological and behavioural results for participants in the Sensorimotor Integration Task 2. Amplitudes of early and late CNV by group at Fz, Cz, and Pz. Standard deviations (SD) provided in parenthesis.

	Control(n = 17)	Pre-HD(n = 19)	Symp-HD(n = 12)
**Behavioural (Tapping)**			
Reaction Time (ms)	750.97 (203.08)	779.11 (243.62)	1306.66 (324.12)^a^ ** ^b^ **
**Electrophysiological (Peak Amplitude/Area)**			
*Fz*			
Peak Early CNV	-4.14 (2.85)	-6.43 (7.49)	-.231 (3.65)
Peak Late CNV	-5.49 (4.94)	-5.35 (3.42)	-.034 (4.90)^a^ ^b^ *
Peak Difference	1.35 (3.53)	-1.07 (5.87)	-.197 (3.21)
Area Early CNV	-1.87 (3.06)	-3.39 (5.13)	1.79 (3.09)
Area Late CNV	-3.89 (4.26)	-3.27 (2.77)	1.61 (5.15)^a^ ^b^ *
*Cz*			
Peak Early CNV	-3.55 (3.32)	-7.23 (6.37)	-.67 (3.65)^b^ *
Peak Late CNV	-8.70 (4.14)	-7.75 (4.81)	-3.68 (6.43)
Peak Difference	5.15 (4.04)	.51 (5.47)a*	3.00 (4.49)
Area Early CNV	-.946 (3.27)	-4.21 (5.03)	2.03 (2.99)^b^ *
Area Late CNV	-6.79 (3.59)	-5.93 (4.56)	-1.73 (6.07)
*Pz*			
Peak Early CNV	-2.31 (2.90)	-5.08 (4.76)	-.163 (4.81)^b^ *
Peak Late CNV	-6.83 (4.21)	-6.99 (5.49)	-1.80 (5.21)^b^ *
Peak Difference	4.53 (4.29)	1.91 (5.07)	1.64 (4.34)
Area Early CNV	.419 (2.77)	-2.30 (4.05)	2.96 (3.80)^b^ *
Area Late CNV	-4.94 (4.03)	-5.60 (6.69)	-.066 (4.94)

‘Peak amplitude’ refers to the maximum negative value in micro volts obtained within the early CNV or late CNV period, with early CNV defined as the period 550–750 ms following stimulus 1, and late CNV defined as the period in the 200 ms prior to stimulus 2. Area refers to the mean amplitude in micro volts during each respective CNV period, obtained separately for each electrode site, and is included to support peak amplitude. Independent samples t-tests used for differences between groups,

* *p* < .05,

** *p* < .01.

^a^ significantly different from controls,

^b^ significantly different from pre-HD.

### Electrophysiological

#### Readiness Potential (RP)—Cued and Self-Paced Tapping (Task 1)

**Fig 1:**
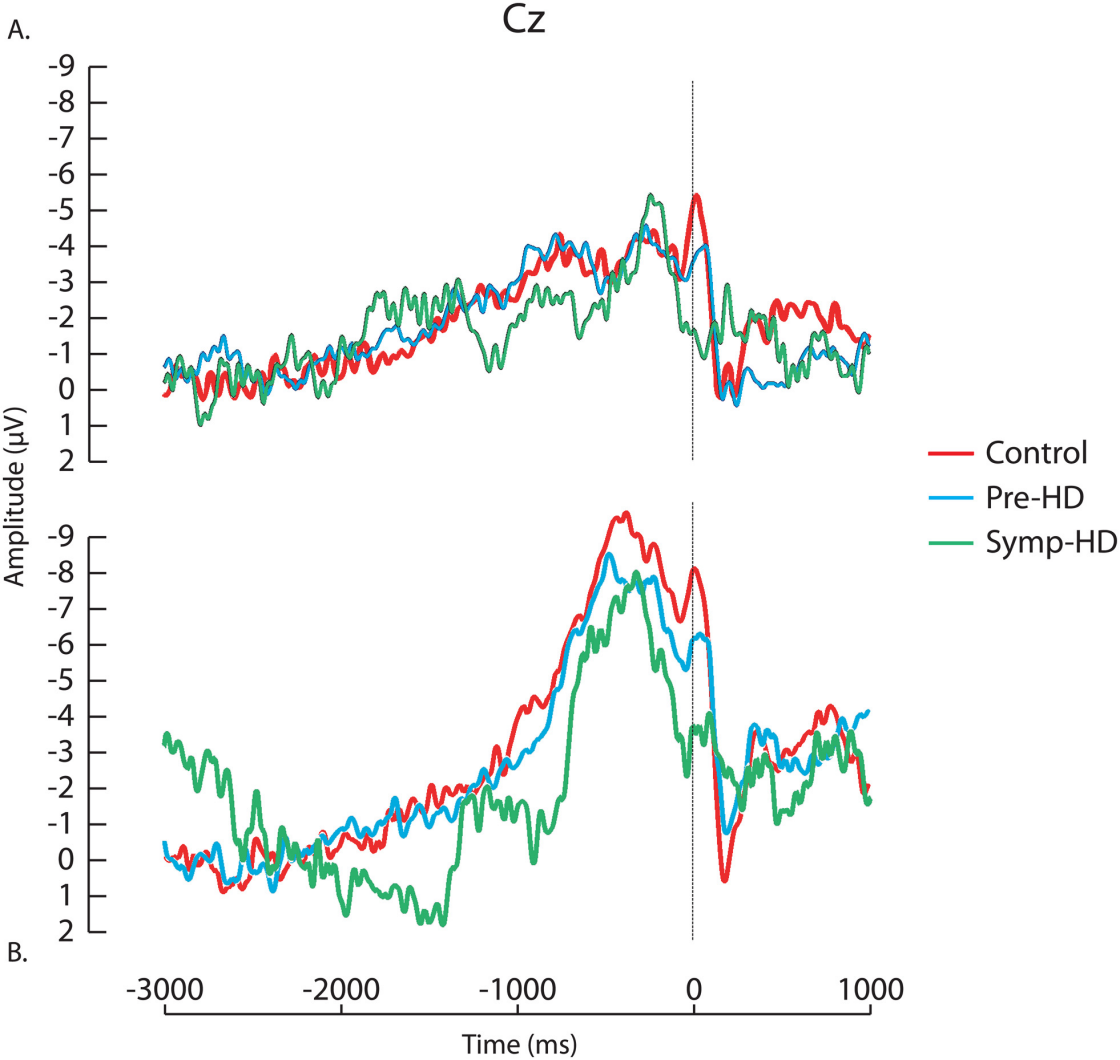
Tapping Task 1 (cued and self-paced Conditions). Grand average Readiness Potential (RP) waveforms are shown at Cz for each of the control, pre-HD and symp-HD groups. (A) cued tapping. (B) Self-paced tapping. Time ‘0’ represents the time of motor response (right index finger button press).

Table 2 displays peak and relative amplitude values and relative latency at Cz; grand average waveforms of the RP are shown in Fig 1. There was a significant main effect of Group for RP relative amplitude (*F* (2, 40) = 10.52, *p* < .001), with larger relative amplitudes for controls (-9.99 *±* 4.58, *p* < .001) and pre-HD (-7.97 ± 3.17, *p* = .009) compared to symp-HD (-3.51 ± 2.30). There was also a significant main effect of Group for relative latency (*F* (2, 40) = 3.35, *p* = .045); post-hoc analyses only identified a trend-level reduction in RP latencies for controls (-160.88 ± 61.35) compared to symp-HD (-209.56 ± 72.40, *p* = .052).

#### Contingent Negative Variation—Sensorimotor Integration (Task 2)

**Fig 2:**
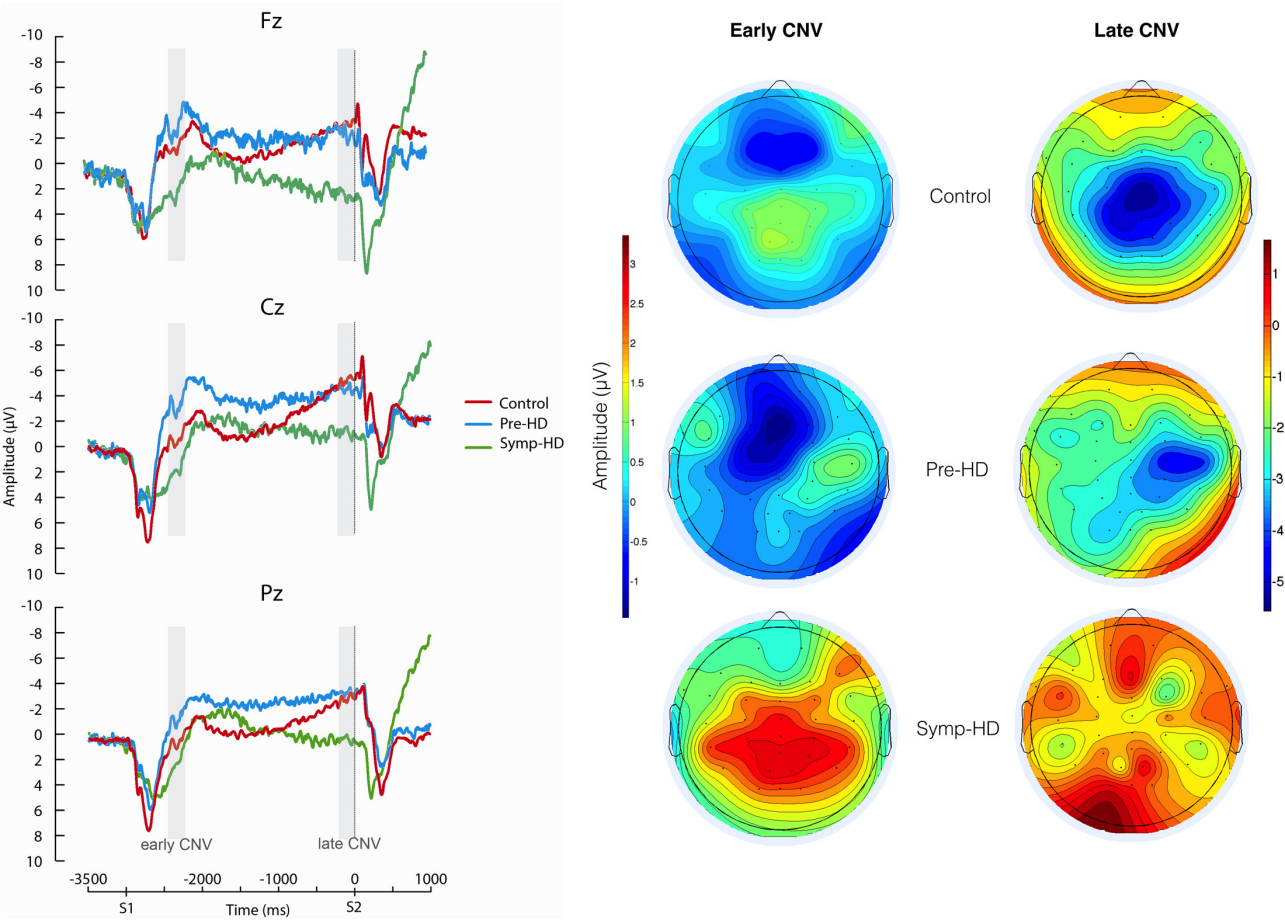
Sensorimotor Integration Task/CNV. Grand average Go/No-Go waveforms at Fz, Cz and Pz across subjects; S1 and S2 are the warning and stimulus onset times respectively. Scalp topography maps represent mean amplitude of the CNV at early and late periods by group. Early CNV refers to the period 550–750 ms following presentation of the warning light (stimulus 1); Late CNV refers to the period 200 ms prior to the onset of the Go/No-Go cue for button press (stimulus 2).

Peak and mean amplitudes of the eCNV and lCNV at Fz, Cz, and Pz for each group separately are shown in Table 3. CNV values at Cz constituted the main analysis, with values at Fz and Pz constituting an exploratory analysis. Grand average waveforms and exploratory topographic maps are provided in Fig 2. Typical grand mean distribution is observed with CNVs maximal at central scalp sites (Cz), and beginning approximately 1500 ms prior to presentation of stimulus 2 (S2). Exploratory topographic maps suggest lateralisation of the early and late CNV in pre-HD.

There was a significant main effect of Group for eCNV peak amplitude (*F* (2, 44) = 4.697, *p* = .014), with symp-HD (-0.67 ± 3.65) amplitudes reduced relative to pre-HD (-7.23 ± 6.37, *p* = .026). No group differences were found for lCNV. However, examination of relative amplitude (early/late peak amplitude difference) revealed a significant main effect of Group (*F* = 4.190, *p* = .022), in which pre-HD relative amplitude (0.51 ± 5.47) was significantly (*p* = .018) smaller than that of controls (5.15 ± 4.04).

For the eCNV, peak amplitude at Pz was significantly different across groups (*F* (2, 44) = 3.637, *p* = .035), with the difference between symp-HD (-0.16 ± 4.81) and pre-HD approaching significance (-5.08 ± 4.76, *p* = .056). In the lCNV, peak amplitude at Fz was significantly different among the groups (*F* = 4.298, *p* = .020); symp-HD (-0.03 ± 4.9) had significantly smaller amplitude than controls (-5.49 ± 4.94, *p* = .028) and pre-HD (-5.35 ± 3.42, *p* = .030). At Pz, peak amplitude was significantly different across groups (*F* = 3.738, *p* = .032); symp-HD were significantly smaller than pre-HD (-1.80 ± 5.21 and -6.99 ± 5.49 respectively, *p* = .040).

#### Correlational Analyses—Sensorimotor Integration (Task 2)

For pre-HD, lCNV peak amplitude at Cz correlated with CAG repeats (.526, *p* = .025). For the symp-HD group, Pz lCNV peak amplitude correlated with CAG repeats (.636, *p* = .026), and DBS (.625, *p* = .030). Peak amplitude for both Fz eCNV (.608, *p* = .036) and lCNV (.645, *p* = .021) correlated with UHDRS motor score.

## Conclusions

This study investigated neural motor and premotor processing during a simple motor task in Huntington’s disease. Symp-HD were significantly differentiated by behavioural measures (slower, more variable taps across tasks) and electrophysiological activity in both motor (smaller relative RP amplitudes) and premotor components (smaller early and late CNV peaks at Cz, and Fz and Pz respectively). Further, the late component at Pz was correlated with established measures of disease progression such as CAG repeats and DBS, supporting a link between aberrant motor processing and clinical degeneration. Consistent with our hypothesis, results indicated group differences between pre-HD and controls for premotor, but not motor measures. No behavioural variable (i.e., reaction time or tapping variability) or electrophysiological activity at time of response (i.e., RP) significantly differentiated pre-HD from controls, suggesting equivalent performance across groups. Importantly, premotor electrophysiological activity (the CNV) significantly differentiated pre-HD from controls, with more widespread electrical activation (Fz, Cz, Pz) and early and elevated preparatory motor activity. Aberrant electrical activity was further suggested by exploratory scalp topography, with partial lateralisation in pre-HD, but not control or symp-HD groups, across the CNV periods. Activation across frontal and parietal regions in pre-HD may suggest a shift in cortical motor control away from primary and secondary motor areas. As there was no performance impairment, we argue that this increased activation may facilitate neural compensation, allowing cognitively intact pre-HD individuals to offset dysfunction [3–5, 11–14].

The RP, a well-established measure of motor activity, began approximately 2 seconds prior to the finger tap, occurring maximally at the vertex (Cz), and was largest in self-paced finger taps, within each group. We used peak-to-peak values to represent the difference in processing between the pre-movement negative peak, and the positive peak occurring after movement. Symp-HD significantly differed from controls and pre-HD groups in relative amplitude of the component, generating motor potentials which were smaller in amplitude in response to the button press. Relative latency also approached significance, with symp-HD responses longer than those of controls, particularly during self-paced tapping. As expected, the RP in pre-HDs did not differ significantly from controls or symp-HD groups. Disruption of the RP in symp-HD, but not pre-HD stages, is an important finding and may reflect increased inefficiency of the motor cortex with more time required to reach maximum amplitudes (which are significantly smaller than controls). Accordingly, this finding was accompanied by significantly slower reaction times in symp-HD. Accordingly, this finding was accompanied by significantly slower reaction times in symp-HD, but not response inhibition inaccuracy (No-Go errors). Breakdown of response inhibition has previously been found in symp and pre-HD [35–36]. The contrary findings to previous studies may be due to differences in our task parameters (e.g. cue prior to Go/No-Go stimuli, no timing feedback), which may interact with these complex processes to suggest maintained response inhibition accuracy in HD despite impairment. However, if response inhibition is intact, but reaction time impaired, this could suggest that costs of maintaining accuracy are also be reflected in inefficient processing of the RP. Inefficient and inaccurate motor execution in symp-HD has been previously suggested by abnormal force, precision, range and velocity during motor tasks [37–38]. The degree of structural degeneration in symp-HD may lead to an inability to recruit additional brain regions as part of a compensation process [14]. Regulation of the motor response itself may be impaired, as symp-HD have shown reduced ipsilateral inhibition and secondary, contralateral activation following normal contralateral MRP activation during a choice reaction task [12]. This could also be related to disruption of hemispheric communication of the basal ganglia; for example, individuals with unilateral basal ganglia lesions demonstrate RPs with a reduced gradient in ipsilateral and contralateral hemispheres following wrist flexion with bilateral presentation [39]. Compensation likely occurs across modalities; recruitment of additional brain regions in maintenance of performance has been previously identified in working memory, with increased, differential prefrontal activation identified in pre-HD in absence of performance detriment [16, 40]. The intactness of the neural motor response in pre-HD, coupled with no difference in reaction times compared with controls, support a likely functional compromise.

For the premotor component (CNV), symp-HD demonstrated significantly smaller peak amplitudes at Cz and Pz compared to the pre-HD group; however, there were no significant differences in the early CNV at Cz between symp-HD and controls. This result is in contrast to Tommaso and colleagues [31], who suggested that early CNV impairment represents an attentional impairment prior to execution of the motor response in symp-HD. We argue against this theory as symp-HD showed no significant difference in number of correct/incorrect responses during Go/No-Go (Task 2) performance, despite significantly slower reaction times. In the late CNV component, symp-HD had significantly reduced peak amplitude at Fz, compared with pre-HD and control groups. Peak amplitude at Pz was also significantly reduced in symp-HD, compared to pre-HD, and positively correlated with CAG repeats and DBS in symp-HD. Tommaso and colleagues [28] found no differences in late CNV, and proposed that this component may become reduced in later stages of the disease. This is plausible given that the prior study had an average illness duration of 2.9 years compared to an estimate of 4.1 years (sample sizes equal; *SD* = 12.96) in the present study. Reduced late CNV has been previously found in Parkinson’s disease and has been associated with dysfunction in the basal ganglia and associated thalamo-cortical circuitry [24], and later in HD as a consequence of further degeneration. Moreover, in symp-HD peak amplitudes of late CNV at Pz were correlated with DBS and CAG repeats, at Fz with UHDRS motor score in, and in pre-HD Cz correlated with CAG repeats. The positive correlations may indicate a link between atypical network recruitment and progressing disease pathology. In both the early and late CNV, exploratory scalp topography suggested positive and aberrant activation in symp-HD, which may support increasing inefficiency of the motor system due to degeneration. However, considerable variance was apparent and likely reflects individual differences in disease progression, duration, and cognitive reserve.

The CNV waveform typically appears as a negative, slow rise slope [24]. Unlike that of controls, the waveform of the pre-HD CNV was not maximal at Cz. Intriguingly, the waveform for pre-HD was higher in amplitude and flattened relative to controls and symp-HD, suggesting early, uniform activation, occurring consistently throughout the preparatory period between the two contingent stimuli. This difference between early and late CNV was significant only for pre-HD. The consistency of this waveform across Fz and Pz suggest a shift in cortical motor control involving the recruitment of additional brain regions. This may be reflected in scalp topography; lateralised activation was evident in both early and late CNV maps for pre-HD. Although not significant, the waveform difference at Cz trended towards significance in symp-HD, producing considerably flatter CNV profiles than control counterparts. This likely reflects more significant and widespread neural dysfunction, which restricts resources and cognitive reserve which could otherwise enable task performance in the context of degeneration. Thus, CNV may represent a sensitive measure of disease progression in pre-HD.

There are a few limitations of the study: the symp-HD group was not age-matched to healthy controls, and were significantly older and more male predominant than controls, as well as displaying more executive dysfunction. However, such findings are typical in a symp-HD group, and we controlled for these factors by including age and gender as covariates. Unfortunately, it is not practicable to partial out the effect of executive dysfunction. As mentioned, considerable variability was evident across individuals within groups in the RP and CNV results (i.e. within pre-HD).

There were also clear differences in the CNV profile between both the early and late peaks for individuals. The decision to include peak to peak values was made after viewing the individual ERPs, not the group averages. When we viewed the group averages our decision was supported; it became evident that simply using measures of peak amplitude could not possibly capture group differences due to variability and the influence of other factors (such as motivation) on amplitude measures. When examining the CNV profile (Fig 2), it is clear that a measure of amplitude for early and late CNV would not distinguish pre-HD from healthy controls, despite clear differences in the profile of activation. We included peak to peak as a value to better capture the degree of activation across the potential, and efficiency of the premotor and motor systems in preparing for the task.

In summary, this study replicated previous findings of no significant behavioural differences during motor task performance in pre-HD compared with controls [10, 13, 41–42]. Importantly, we identified irregular premotor processing (CNV) in pre-HD, which involved more widespread activation during sensorimotor integration, despite intact motor processing (RP). This may support theory that during the early premanifest stages the brain may undergo a process of maintenance of cortical motor control, which involves recruitment of additional brain regions and networks to preserve function. Such compensatory mechanisms appear to be more heavily recruited during more cognitively demanding tasks. Performance intactness in the pre-HD group suggests compensation may be a reciprocal feedback process, employed to counterbalance dysfunction in response to unique processing demands. Overall, recruitment of ancillary circuitry in preservation of motor functions may represent a sensitive functional biomarker of disease progression in HD. Future studies in this area using techniques such as functional MRI, magnetoencephalography or standardized low resolution brain electromagnetic tomography may be able to further elucidate functional connectivity during neural premotor processing, such as the contributions of neostriatal networks.
